# *In vitro* and *in vivo* antidermatophytic activity of the dichloromethane-methanol (1:1 v/v) extract from the stem bark of *Polyscias fulva* Hiern (Araliaceae)

**DOI:** 10.1186/1472-6882-13-95

**Published:** 2013-05-06

**Authors:** Guy Sedar Singor Njateng, Donatien Gatsing, Raymond Simplice Mouokeu, Paul Keilah Lunga, Jules-Roger Kuiate

**Affiliations:** 1 Laboratory of Microbiology and Antimicrobial Substances, Faculty of Science, University of Dschang, P.O. Box 67, Dschang, Cameroon; 2 Institute of Fisheries and aquatic sciences, University of Douala-Cameroon, PO box 2701, Douala, Cameroon; 3 Laboratory of Phytobiochemistry and Medicinal Plants Study, Faculty of Science, University of Yaoundé 1, P.O. Box 812, Yaoundé, Cameroon

**Keywords:** Antidermatophytic activity, Polyscias fulva, Extract-oil formulation, Irritation test, Primary irritation index, Guinea pigs

## Abstract

**Background:**

During the last decades, the number of people suffering from dermatophytoses has seriously increased, mainly due to the development of resistant strains of microorganisms to a range of formally efficient antibiotics. *Polyscias fulva,* a medium size tree which grows in the West Region of Cameroon is traditionally used for local application against dermatoses and orally against venereal infections. The dichloromethane-methanol (1:1 v/v) extract from the stem bark of *Polyscias fulva* was evaluated for its *in vitro* and *in vivo* antifungal activities.

**Methods:**

The plant extract was prepared by maceration of its stem bark powder in CH_2_Cl_2_-MeOH (1:1 v/v). The extract obtained was successively partitioned in hexane, ethyl acetate and n-butanol. Phytochemical screening was performed using standard methods. *In vitro* antidermatophytic activity was assayed by the well diffusion and broth microdilution methods. The degree of dermal irritation of the crude extract was determined in guinea pigs using the occluded dermal irritation test method. The *in vivo* antidermatophytic activity of the extract-oil formulation (1.25, 2.5 and 5% w/w concentrations) was evaluated using *Trichophyton mentagrophytes-*induced dermatophytosis in a guinea pigs model.

**Results:**

Phytochemical screening indicated that, the crude extract, ethyl acetate, n-butanol and residue fractions contain in general saponins, tannins, alkaloids, anthraquinones and phenols while the hexane fraction contains only alkaloids. The ethyl-acetate, n-butanol and residue fractions displayed higher antifungal activities (MIC = 0.125-0.5 mg.mL^-1^) against eight dermatophytes as compared to the crude extract (MIC = 0.5-1 mg.mL^-1^). This latter appeared to have slight perceptible erythema effects on guinea pigs as the primary irritation index (PII) was calculated to be 0.54. *In vivo,* the antidermatophytic activities of the extract-oil formulations were dose-dependent. Griseofulvin-oil 5% at 0.01 g/kg and formulated extract-oil (5%) at 0.1 g/kg eradicated the microbial infection after thirteen and fourteen days of daily treatment respectively.

**Conclusions:**

The results of preclinical *in vitro* and *in vivo* evaluations indicate that the extract-oil formulation at 5% may constitute an alternative means to alleviate fungal infections caused by dermatophytes.

## Background

Dermatophytic infections, often referred to as ringworm and tinea are known to cause universal affliction of humans and their domestic animals. Dermatophytes colonize the skin, nails, and hair of human populations together with similar infections on animal hides, hooves, feathers, beaks, and other dermal appendages [1]. Dermatophytes thus affect keratinous tissues of humans and other vertebrates, causing superficial infections. These microorganisms belong to three genera: *Trichophyton*, *Microsporum* and *Epidermophyton*[1]. Dermatophytoses generally comply with topical antifungal therapy, although local therapy may be inappropriate for extensive infections or for infections affecting the nails or scalp. In recent years, a number of antifungal agents have been introduced into clinical practice including griseofulvin, terbinafine, itraconazole, fluconazole, and more recently voriconazole [1,2]. However, their spectrum of action is narrow and also many of them have adverse side effects [3,4]. The treatment of these infections is often long and expensive, and there have been increasing reports of antifungal resistance [5]. Thus, there is a need for the search for new sources of antidermatophytic substances. Herbal medicines have been widely used and form an integral part of primary health care in many countries [6], and may constitute a reservoir of new antifungal substances. According to World Health Oganisation, medicinal plants would be the best source of a variety of drugs [7]. About 80% of the total population in developing countries relies on traditional medicines based on plant products [8]. Unfortunately, their traditional use remains empirical, doses and durations of treatment for example are not clearly defined. There is therefore need for chemical and pharmacological studies that may help to achieve the standardization of such treatment. This explains why numerous studies have been conducted on various medicinal plant extracts with the hope of discovering new and more efficient antifungal compounds.

Among these plants is *Polyscias fulva,* a medium size tree which grows in the tropical forests of West and Central Africa, and is traditionally used to treat malaria and mental illness [9]. In Cameroon, a decoction of the stem bark of *Polyscias fulva* bark is traditionally taken orally against venereal infections [10]. Furthermore, its stems and leaves are pounded and used for local application against dermatoses. Phytochemical analyses revealed the presence of triterpenes glycosides [11] and others compounds like Lichexantone, 3-o-β-D-glucopyranosyl-stigmasterol, 3-α-L-arabino-pyranosyl-hederagenin (cauloside A), α-hederin and kalopanax-saponin B [12] from the aerial part of the plant. Based on traditional uses of this plant, we believe that the extract could possess antidermatophytic proprieties and an oil-extract formulation may be effective in the treatment of dermatophytic infection. Several similar studies have been conducted and showed the relative effectiveness of such preparations. For example, we can mention the work on the essential oil of Chenopodium ambrosioides [13], Zataria multiflora [14] and Eucalyptus camaldulensis [15]. The main difficulty of such a study is the induction of dermatophytosis in a suitable animal model.

To the best of our knowledge, no work exists on the antidermatophytic activity of this plant. Thus, the present study was undertaken to evaluate the *in vitro* and *in vivo* antifungal activities of the dichloromethane-methanol (1:1v/v) extract from the stem bark of *Polyscias fulva*.

## Methods

### Plant material

The stem bark of *Polyscias fulva* (Hiern) was collected in April 2008 at Bazou (Nde Division, West Region, Cameroon). Botanical identification was done at the Cameroon National Herbarium in Yaoundé by Mr Tadjouteu Fulber and where a voucher specimen was kept under the reference number 43546/HNC.

### Microorganisms

Tests were performed on eight dermatophytes: four clinical isolates including *Microsporum audouinii* from the “Centre Pasteur” of Yaoundé, *Trichophyton rubrum* from the “Centre Pasteur” of Paris, *Trichophyton ajelloi and Trichophyton equinum from* “Ecole Nationale Vétérinaire d’Aford” in France; four strains including *Trichophyton mentagrophytes* (E 1425), *Trichophyton terrestre* (E 1501), *Trichophyton Microsporum gypseum* (E 1420) and *Epidermophyton floccosum* (E 1423), all from “Ecole Nationale Vétérinaire d’Aford” in France. The strains have been maintained in the refrigerator at 4°C on agar slant as previously described [16].

### Animals

The dermal irritation test and the *in vivo* Antidermatophytic activity of the crude extract were performed using adult guinea pigs of both sexes (400 ± 50 g) bred in the Animal House of the Department of Biochemistry, University of Dschang, Cameroon. The animals were fed with standard guinea pig feed and water *ad libitum* throughout the experimental period. Animals were exposed to natural room temperature (22±2°C) and were handled according to standard protocols for the use of laboratory animals [17,18]. Animals were randomly selected and caged individually or in a group, depending on the type of assay, for 5 days prior to the start of the test for acclimatization to the test conditions. They were also randomly assigned to control and test groups.

### Preparation of plant extract and fractionnation

The stem barks of *P. fulva* were dried at room temperature (20–24°C) for fifteen days and powdered to coarse particles. Four kilograms of powder were macerated in 10 L of the dichloromethane-methanol (Merck) (1:1 v/v) mixture for two days with frequent stirring and this process was repeated twice on the residue. After filtration, using Wathmann filter paper N° 1, the filtrate was evaporated to dryness under vacuum at 45°C using a rotary evaporator (Büchi R200). The obtained crude extract (263 g) obtained was then partitioned successively in hexane, ethyl acetate and n-butanol to obtain the hexane (27.25 g), ethyl acetate (23.77 g), n-butanol (16 g) and residue (195.98 g) fractions after solvent evaporation. The percentages of crude extract and fractions are reported with respect to the plant material and crude extract respectively. The number of compounds in the crude extract and fractions were estimated based on their thin-layer chromatography (TLC) profiles. Mixtures of ethyl acetate-methanol were used as elution as follow: 8:2 for the crude extract and ethyl acetate fraction; 10:1 for hexane fraction, 9:3 for n-butanol fraction and 6:4 for the residue fraction. Spots and bands were visualized by UV radiation (254 and 366 nm) and by spraying with 50% (v/v) sulphuric acid reagent followed by heating at 100°C.

### Phytochemical screening

The phytochemical analysis of the crude extract and its fractions were carried out using standard methods [19]. The plant material was screened for the presence of different classes of compounds including alkaloids, flavonoids, sterols, triterpenes, coumarins, anthraquinones, tannins, anthocyanins, saponins and phenols.

### *In vitro* Antidermatophytic activity

#### Preparation of dermatophyte inocula

The spore suspensions used for the inoculation were prepared from a 15 days old cultures on Sabouraud Dextrose Agar (Conda, Madrid, Spain) culture medium. The culture surfaces were gently scraped and placed in test tubes containing 10 mL of sterile saline; then homogenized by end-to-end mixing for 5 minutes and filtered. The absorbances of the spore suspensions (filtrates) were read at 530 nm and adjusted with sterile distilled water between 0.15 and 0.17 to match 0.6 × 10^6^ -1.4 × 10^6^ CFU.mL^-1^[20].

#### Antidermatophytic activity using the well diffusion method

The well diffusion test was carried out as previously described [21]. Briefly, stock solutions of the extracts (crude extract and fractions) were prepared in 5% v/v aqueous dimethyl sulphoxide (DMSO, Fisher Chemicals) at a concentration of 100 mg.mL^-1^. Glass bottles, each containing 19.8 mL of sterile, matten Sabouraud Dextrose Agar medium were maintained in a water bath set at 40°C (to prevent solidification of the medium) and then inoculated aseptically with 0.2 mL of a given spore suspension followed by thorough mixing. Each 20 mL mixture was poured into a sterile Petri dishe (90 mm of diameter) and allowed to solidify. A wells of 6 mm in diameter was then made in the middle of SDA plate and filled with 50 μl of an extracts prepared at 50 mg.mL^-1^. The inoculated plates were then incubated at 30°C for five days. The susceptibility was recorded by measuring the diameter of the clear zone of growth inhibition on the agar surface around each well. All the experiments were repeated thrice: Griseofulvin (Merck, Darmstadt, Germany) at 10 μg/well was used as positive control while the dilution solution (5% (v/v) aqueous DMSO) served as negative control.

#### Antidermatophytic activity using broth microdilution method

The broth microdilution method [22] was used to determine the minimum inhibitory concentration (MIC) and minimum fungicidal concentration (MFC) of the tested substances using 96 well microplates (Nunclon, Roskilde, Danmark). Two-fold serial dilutions of the extract (Concentration range: 8–0.0625 mg.mL^-1^), fractions (Concentration range: 1–0.0078 mg.mL^-1^) and reference drug (Concentration range: 0.1-0.00078 mg.mL^-1^) were performed in Sabouraud Dextrose Broth (SDB) in a total volume of 200 μL per well. Each well contained 100 μL of the test substance at a particular concentration, and 100 μL of the fungal spore suspension corresponding to approximately 10^4^ CFU.mL^-1^. The microwell plates were covered and incubated at 30°C for five days on a plate shaker (Flow Laboratory, Germany) at 300 rpm. Fungal growth in each well was determined by observing and comparing the turbidity of the test wells to that of the positive and negative controls. The MIC was the lowest concentration of the test substance that prevented visible growth of the microorganisms.

The minimum fungicidal concentration (MFC) values were determined by subculturing 50 μL aliquots of the preparations, which did not show any visible growth of the micro-organisms during MIC determinations. These preparations were further incubated at 30°C for five days on the plate shaker at 300 rpm. Microbial growth in each well was determined by observing and comparing the turbidity of test wells to that of the positive and negative controls. The MFC was the lowest concentration of the test substances that prevented visible growth of the microorganisms in the sub-cultures. For every experiment, sterility controls (5% v/v aqueous DMSO, medium, inocula and griseofulvin) were included. All the experiments were performed in triplicates.

#### *In vivo* study

### Ethical statement

All studies involving animals were conducted according to the ethical guidelines of the Committee for Control and Supervision of Experiments on Animals (Registration no. 173/CPCSEA, dated 28 January, 2000), Government of India, on the use of animals for scientific research.

### Skin irritation test

The degree of dermal irritation of the dichloromethane-methanol (1:1v/v) extract from the stem bark of *Polyscias fulva* was determined in guinea pigs using the occluded dermal irritation test method [23]. Twelve guinea pigs (six males and six females) were used and each animal served as its own control. They were divided into two groups (group 1 and group 2) of six animals each (3 males and 3 females) and treated with water-moistened extract and oil-moistened extract respectively. On day 0, fur was shaved from the back of each animal, about 6 cm^2^ on both sides. The left side served as a negative control, while the right one served as a test site. The guinea pigs were caged individually for 24 h. On day 1, the extract (0.5 g) moistened with distilled water was evenly applied to the test sites of group 1 animals and the skin was covered with a gauze patch, plastic sheet and a non-irritant adhesive plaster. The control sites were treated with distilled water and covered as above. The animals of group 2 were treated with oil-moistened extract in the same manner as above. Palm kernel oil was used and also served as the negative control for the group 2 experiment.

After 24 h of exposure, the coverings were removed and the test site rinsed with distilled water and dried. The animals were examined for the presence of erythema and edema according to the Draize dermal irritation scoring system (0, no erythema or no edema; 1, barely perceptible erythema or edema; 2, well defined erythema or slight edema; 3, moderate to severe erythema or moderate edema; 4, severe erythema or edema) at grading intervals of 1, 24, 48 and 72 h [24].

The primary irritation index (PII) was calculated by dividing the sum of erythema and edema scores of the grading intervals by the number of grading intervals (4). The extract was then classified according to the Draize method of classification using the PII scoring method as mildly irritant (PII< 2), moderately irritant (2 ≤PII≤ 5), and severely irritant (PII>5).

### *In vivo* antidermatophytic activity

This experiment was carried out using the crude extract. The fungal inoculum was prepared as described above in the section on *in vitro* antidermatophytic activity.

#### Dermal infection of animals

Both male and female guinea pigs (350-450 g) were used. The fur from the back of each guinea pig was shaved (approximately 16 cm^2^) and 50 μL of a suspension of *T. Mentagrophytes* spores was inoculated to a surface of about 4 cm^2^ area (previously lightly abraded with sandpaper) within the shaved zone [20,25]. Animals were randomly assigned to different treatment groups. For each sex, there were seven groups of three animals each. Evidence of infection was revealed by direct observation of the infected area, followed by agar culture of scrapings from the area, and microscopic observation of the resulting fungi from the scrapings.

#### Treatment of infected animals

Animals were treated by dermal application of the test substance which started on the sixth day after animal infection and was continued daily (each morning). All animals except those of group 1 were infected and treated (Table 1).

**Table 1:** Infection and treatment of guinea pigs

**Group**	**Group type**	**Infection**	**Treatment**
1	Simple control	No	No
2	Negative control	Yes	No
3	Palm kernel oil control	Yes	0.1 g/kg of palm kernel oil (the vehicle)
4	Positive control	Yes	0.01 g/kg of griseofulvin-oil at 5%
5	Test	Yes	0.1 g/kg of extract-oil at 1.25% (w/w)
6	Test	Yes	0.1 g/kg of extract-oil at 2.5% (w/w)
7	Test	Yes	0.1 g/kg of extract-oil at 5% (w/w)

#### Clinical surveillance of treatment

Clinical response was monitored by assigning a score between 0 and 4 to each infection site: 1 (for flakinsg skin with mild inflammation); 2 (for slight lesions with moderate inflammation); 3 (for moderate lesions with severe inflammation) and 4 (for severe inflammation plus crust formation). The results were expressed as the percentage reduction in mean lesion score at the time when placebo-treated (group 3) animals showed maximal severity (lesion score 4) [26].

The degree to which the animals complied with treatment was estimated by culturing skin scrapings and fur on Sabouraud Dextrose Agar supplemented with actidione (Conda), for recovery of viable *T. Mentagrophytes* cells. These scrapings and fur were collected from the active border of the infection site every two days [27]. The cultures were incubated for 15 days at 28°C. The results were recorded in terms of percentage culture recovery of *T. Mentagrophytes* of the infected site [14].

$$\%\;\mathrm{Culture}\;\mathrm{recovery}=\frac{\begin{array}{r} \mathrm{Total number of sites showing}\\ \mathrm{presence of dermatophytes} \end{array}}{\mathrm{Total number of infection sites}}\times 100$$

### Statistical analysis

Data on irritation are presented as visual scores based on Draize method of erythema and edema-grading system and PII was calculated, whereas the inhibition diameters of crude extract and fractions were expressed as the mean ± standard deviation. The Waller-Duncan test was used to compare the means for the different substances tested, at 5% significance level.

## Results

### Phytochemical screening of extract and fractions

The phytochemical screening revealed that all the classes of compounds (saponins, tannins, alkaloids, anthraquinone and phenols) present in the crude extract were found in the ethyl acetate fraction (Table 2). The hexane fraction had only alkaloids. The n-butanol and residue fractions had almost the same composition except that anthraquinones were present in n-butanol fraction, while alkaloids were in the residual fraction.

**Table 2:** **Physical characteristics and phytochemical analysis of crude extract of *Polyscias fulva *stem bark and its fractions**

**Extracts**	**Aspect/colour**	**Yield (% )**	**Number of spots in TLC**	**Positive tests for**	**Negative tests for**
Crude extract	Darkish-brown/paste	6.58	9	Saponins, Tannins, Alkaloids’, Anthraquinone and Phenols	Flavonoïd, Triterpen, Sterol, Anthocyanin and Coumarin.
Hexane fraction	Greyish-brown/paste	10.36	6	Alkaloids	Flavonoïd, Triterpen, Sterol, Anthocyanin, Coumarin, Saponins, Tannins, Anthraquinone and Phenols.
Ethyl acetate fraction	Darkish-brown/paste	9.04	7	Saponins, Tannins, Alkaloids’, Anthraquinone and Phenols	Flavonoïd, Triterpen, Sterol, Anthocyanin and Coumarin.
n-Butanol fraction	Brown/paste	6.08	5	Saponins, Tannins, Alkaloids’ and Phenols	Flavonoïd, Triterpen, Sterol, Anthocyanin, Coumarin and Anthraquinone.
Residue fraction	Darkish-brown/paste	74.52	3	Saponins, Tannins, Anthraquinone and Phenols	Flavonoïd, Triterpen, Sterol, Anthocyanin, Coumarin and Alkaloids.

### *In vitro* antidermatophytic assay

As revealed by the well diffusion method, the n-hexane fraction was almost inactive on all the tested microorganisms except *E. floccosum*. All the microorganisms appeared to be sensitive to the active substances with *T. mentagrophytes*, *T. ajelloi* and *M. audouinii* being the most sensitive (Table 3). Fractionation did not significantly increase antidermatophytic activities. These results were globally confirmed by the microdilution method for determination of MIC and MFC (Table 4), although on some species, fractions were more active. The MFC/MIC ratios were less than or equal to 2, indicating a possible fungicidal effect of the extract and its fractions.

**Table 3:** **Diameter of the inhibition zones (mm) of CH**_**2**_**Cl**_**2**_**-MeOH (1:1v/v) crude extract and fractions of the stem bark of *Polyscias fulva***

**Microorganisms**	**Diameter of inhibition zones ± Standard deviation (mm)**					
	**Crude extract**	**Hexane fraction**	**Ethyl acetate fraction**	**n-butanol fraction**	**Residue fraction**	**Griseofulvin (10 μg)**
*T. mentagrophytes*	28.0 ± 0.5^b^	0,0 ± 0.0^a^	29.0 ±0.5^c^	29.0 ±0.5^c^	28.1 ±0.2^b^	34.75±0.25^d^
*T. ajelloi*	28.0 ± 0.4^b^	7.0 ± 0.3^a^	28.5±0.5^b^	28.5±0.5^b^	28.0 ± 0.2^b^	30.5±0.5^c^
*T. rubrum*	24.0 ± 0.4^c^	0.0 ± 0.0^a^	24.5±0.5^c^	24.4±0.2^c^	23.0 ±0.4^b^	30±0.5^d^
*T. equinum*	25.4 ± 0.4^c^	0.0 ± 0.0^a^	25.4±0.4^c^	25.0 ±0.5^c^	24.5±0.5^c^	27.5±0.5^d^
*T. terrestre*	25.0 ± 0.5^c^	0.0 ± 0.0^a^	22.0 ±0.3^c^	21.0 ±0.3^b^	22.0 ±0.5^c^	35.83±0.28^e^
*E. flocosum*	21.5 ± 0.5^b^	20.0 ± 0.5^a^	23.0 ±0.5^c^	23.0 ±0.5^c^	22.0 ±0.5^b^	35±0.5^d^
*M. gypseum*	20.3 ± 0.3^b^	0.0 ± 0.0^a^	20.2±0.2^b^	21.0 ±0.3^c^	21.0 ±0.5^c^	34.7±0.20^d^
*M. audouinii*	28.1 ± 0.3^b^	0.0 ± 0.0^a^	29.0 ±0.5^c^	29.1 ±0.2^c^	28.1±0.3^b^	34.75±0.25^d^

The results are the mean values of triplicate tests. Across the line, values affected by the same letter superscript are not significantly different (test of Waller-Duncan (p > 0.05).

**Table 4:** **Minimum Inhibitory Concentration (MIC in mg.mL**^**-1**^**) and Minimum Fungicidal Concentration (MFC in mg.mL**^**-1**^**) of CH**_**2**_**Cl**_**2**_**-MeOH (1:1v/v) extract and fractions of the stem bark of *Polyscias fulva***

**Microorganisms**	**Crude extract**		**Hexane fraction**		**Ethyl acetate fraction**		**n-butanol fraction**		**Residue fraction**		**Griseofulvin (10 μg)**	
	**MIC**	**MFC**	**MIC**	**MFC**	**MIC**	**MFC**	**MIC**	**MFC**	**MIC**	**MFC**	**MIC**	**MFC**
*T. mentagrophytes*	0.25	0.25	4	4	0.125	0.125	0.125	0.125	0.125	0.125	0.00078	0.00078
*T. ajelloi*	0.25	0.25	2	2	0.25	0.5	0.125	0.125	0.125	0.5	0.0003	0.0003
*T. rubrum*	0.5	0.5	8	’8	0.25	0.25	0.25	0.25	0.5	0.5	0.0003	0.0003
*T. equinum*	0.5	0.5	4	8	0.5	0.5	0.25	0.25	0.25	0.25	0.0003	0.0003
*T. terrestre*	1	1	8	’8	0.5	0.5	0.25	0.25	0.25	0.25	0.05	0.1
*E. flocosum*	1	1	1	1	0.125	0.25	0.25	0.5	0.25	0.5	0.0003	0.0003
*M. gypseum*	1	1	8	’8	0.5	0.5	0.25	0.25	0.125	0.25	0.0015	0.0015
*M. audouinii*	0.5	0.5	4	8	0.125	0.25	0.25	0.25	0.125	0.5	0.00078	0.00078

### *In vivo* Antidermatophytic assay

**Figure 1:**
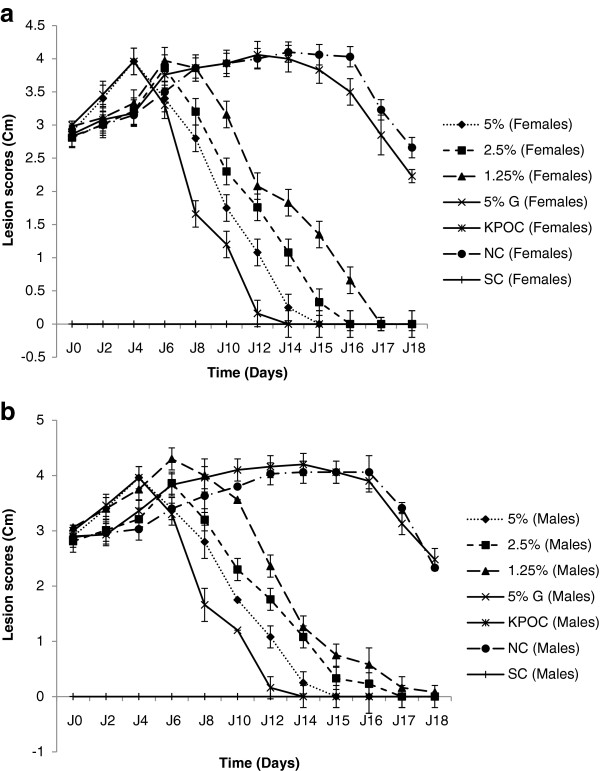
**Evolution of lesion scores during treatment of infected guinea pigs: females (1a) and males (1b).** G: Griseofulvin-oil; KPOC: Palm Kernel oil control; NC: Negative control; SC: Simple control (Non infected or non treated).

**Figure 2:**
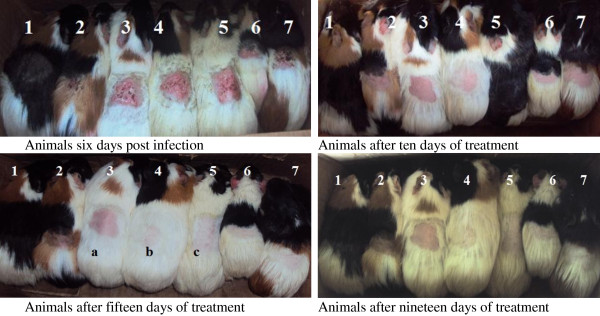
**Comparative illustration of the evolution of the infection during treatment of infected guinea pigs with *P. fulva *extract-oil.** 1: Uninfected animal. 2: Infected untreated animal. 3: Infected animal treated with 0.1 g/kg of palm Kernel oil. 4: Infected animal treated with 0.01 g/kg of griseofulvin-oil at 5%. 5: Infected animal treated with 0.1 g/kg of extract-oil at 1.25%. 6: Infected animal treated with 0.1 g/kg of extract-oil at 2.5%. 7: Infected animal treated with 0.1 g/kg of extract-oil at 5%.

**Figure 3:**
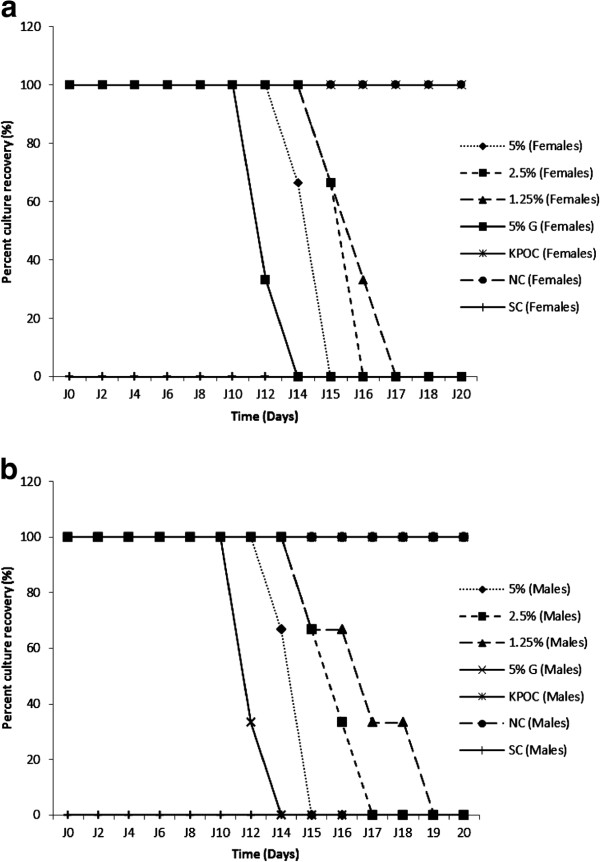
**Evolution of the percent culture recovery during treatment of infected guinea pigs: females (3a) and males (3b).** G: Griseofulvin-oil; KPOC: Palm Kernel oil control; NC: Negative control; SC: Simple control (Non infected or non treated).

Only infected animals were used in this assay. Up to the sixth day of treatment, lesions increased continuously in all the groups. From the 8^th^ day, the intensity of lesions started dropping in the treated groups in a dose-dependent manner (Figures 1 and 2). The Griseofulvin-oil (5%) and extract-oil (5%) mixtures had comparable effects, eradicating surface lesions after 13 and 14 days of treatment respectively. However, the other doses gradually overcome the infection from the 17^th^ day of treatment. Simultaneously, lesions continued to increase in the untreated groups (negative control and the vehicle-treated group), indicating the non-therapeutic effect of the vehicle. These results corroborate the percentage culture recovery experiment as there were no more fungal cells at the infection sites from the 14, 15, 17 and 19^th^ days of treatment for griseofulvin-oil (5%), extract-oil at 5%, 2,5% and 1,25% respectively (Figure 3).

### Skin irritation test

**Figure 4:**
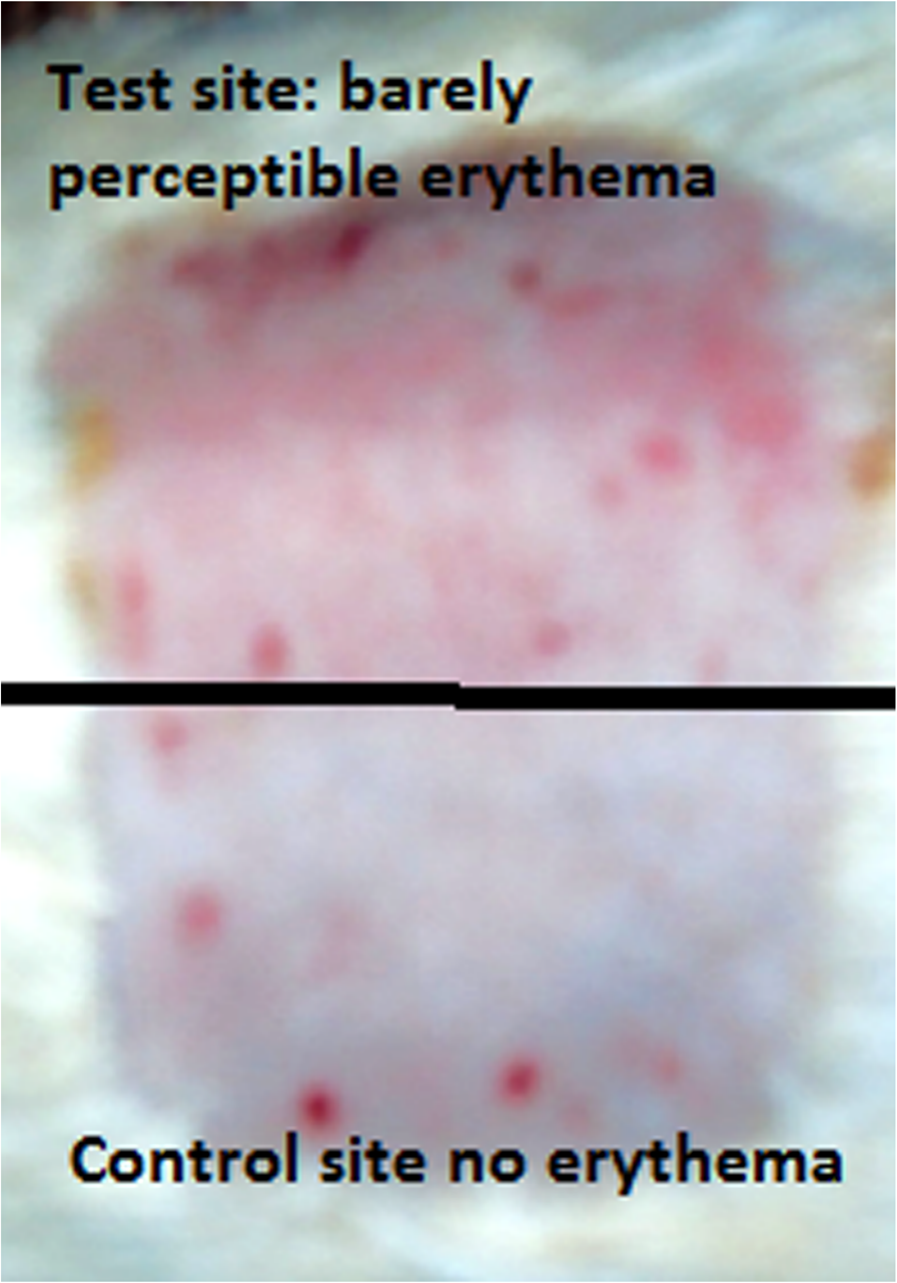
**Illustration of the irritant effect of *Polyscias fulva *extract moistened with distilled water.**

**Figure 5:**
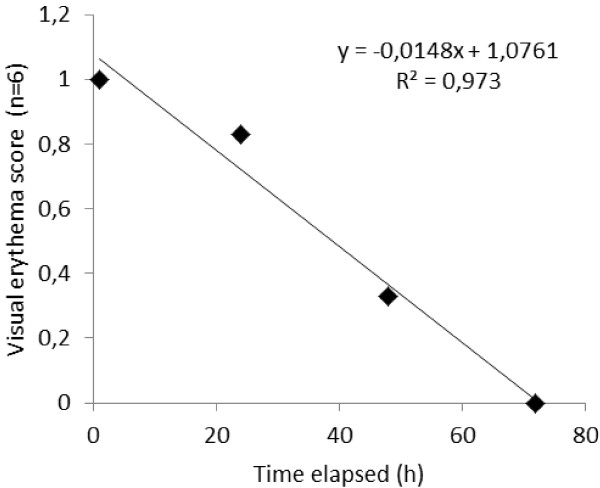
**Degree of irritation and it trends of change since opening of the patch 24 h after dermal application of 0.5 g of dichloromethane-methanol (1:1v/v) extract from the stem bark of *Polyscias fulva.***

Group 1 animals did not show observable edema while barely perceptible erythema effects (PII = 0.54) were evident in guinea pigs of this group (Figure 4). Neither visible erythema nor edema were observed in the guinea pigs of group 2 (PII = 0.00). These results indicate that the use of palm kernel oil as a vehicle is preferable to water. The time course of recovery from irritation is depicted in Figure 5. Scores of erythema following 1 and 2 h after the opening of the patch were similar in intensity. However, the erythema score had sharply decreased and came to zero at the 72 h reading in group 1 (Figure 5). There was no erythema or edema observed on the control sites of all guinea pigs.

## Discussion

### Antidermatophytic activity

Dermatophytes are closely related fungi which induce dermatophytosis. Treatment with topical fungicidal allylamines is more efficient than with fungistatic azoles [28]. Although not life threatening, dermatophytosis are difficult to eradicate, particularly in low income countries, due to the non-accessibility to efficient drugs and ignorance. In these countries, these diseases are mostly treated using medicinal plants. Herbal medicines have been important sources of bioactive substances belonging to diverse chemical groups [29]. The phytochemical screening of the crude extract of *P. fulva* and its fractions revealed the presence of saponins, tannins, alkaloids, anthraquinone and phenols that can explain their antidermatophytic activities. Individual activities of these compounds have been demonstrated [29]. Also, from our previous work, the antidermatophytic activities of triterpenes [30], essential oil of *Ageratum houstonianum*[31] and the volatile fraction of *Cupressus lusitanica*, constituted of monoterpens, sesquiterpens and triterpens [32], were reported. The n-hexane fraction was almost inactive, indicating that the active principle may not belong to the family of alkaloid.

Trichophytosis induced in guinea pigs is a well-established predictive model for testing topical antifungal agents [25]. This model was used in this study and permitted to show that the crude extract of *P. fulva* possesses *in vivo* antifungal activity against a virulent strain of *T. mentagrophytes*. Irrespective of sex, the extract-oil formulation at 5% was able to cure infected animals after fourteen days of treatment while griseofulvin produced the same effect after thirteen days. These results show that, this formulation can be used as a substitute or an alternative to the use of griseofulvin to cure dermatophytosis. It can therefore be a potent candidate for the elaboration of a phytomedicine. Similar results have been reported on the antidermatophytic action of a 1% oil-petroleum gelly formulation of the essential oil of *Chenopodium ambroisoides* which cured *Trichophyton mentagrophytes-*induced dermatophytosis in guinea pigs in 15 days [13]. Also similar effects have been reported on the extract-cream formulation of *Zataria multiflora*[14] and *Eucalyptus camaldulensis*[15].

In dermatophytic infections as observed in this study, inflammatory responses are usually characterized by a greater degree of redness and scaling at the edges of the lesions, or occasionally, blister formations [28]. This is often accompanied by mild irritation though, could be more severe with some types of fungal infection. During inflammation, reactive oxygen species and free radicals with many physiological and pharmacological adverse effects, including skin irritation are produced [33]. The phenols and tannins, identified in the *P. fulva* extract, are known for their ability to protect cells against reactive oxygen species and free radical-induced toxicity [34].

### Skin irritation test

The irritation test revealed a negligible irritant effect of the extract at a high dose (0.5 g) on guinea pig’s skin. The low level of erythema observed quickly decreased after the opening of the occlusion. This can be attributed to the occlusion effect of the extract; a phenomenon known to boost phase I or the vascular event of an inflammatory response [35]. Occurrence of phase II or the cellular event of the inflammatory process was not evident in our study since there were no observable changes in the skin morphology of the guinea pigs. This confirms the assertion that the extract would have only a negligible irritation potential [36,37]. Similar results have been reported on the skin irritation test of an 80% methanol extract of the leaf of *Dodonaea viscose* in rabbits where the primary irritation index was 0.45 [35]. The fact that neither erythema nor edema persisted in the guinea pigs could lend support to the fact that this plant extract is safe at the indicated dose. Also the absence of these clinical signs in the palm kernel oil-treated group suggests that use of this oil as a vehicle for administering this extract, is safe.

## Conclusion

This study demonstrates that the crude dichloromethane-methanol (1:1 v/v) extract from the stem bark of *Polyscias fulva* possesses antidermatophytic properties without irritation effects. These results may justify the use of *Polyscias fulva* in the traditional medicine to cure dermatoses. Besides, palm kernel oil has been demonstrated to be a good vehicle. The oil-extract of *Polyscias fulva* may be a possible candidate for the standardization of an antidermatophytic phytomedicine or for the isolation of pharmacologically active antifungal agents.

## Competing interests

The authors declare that they have no competing interests.

## Authors’ contributions

GSSN is the field investigator and have drafted the manuscript. DG supervised the work and contributed to drafting the manuscript. JRK designed the study, supervised the work and finalized the manuscript. RSM contributed to the phytochemical studies. PKL contributed to the field work and corrected the language of the manuscript. All authors read and approved the final manuscript.

## Pre-publication history

The pre-publication history for this paper can be accessed here:

http://www.biomedcentral.com/1472-6882/13/95/prepub
